# ZIKV infection regulates inflammasomes pathway for replication in monocytes

**DOI:** 10.1038/s41598-017-16072-3

**Published:** 2017-11-22

**Authors:** S. F. Khaiboullina, T. Uppal, R. Sarkar, A. Gorzalski, S. St Jeor, S. C. Verma

**Affiliations:** 1 Department of Microbiology and Immunology, University of Nevada, Reno School of Medicine, Reno, NV USA; 2 Kazan Federal University, Kazan, Russian Federation; 3 Genquest LLC, Sparks, NV USA

## Abstract

ZIKV causes microcephaly by crossing the placental barrier, however, the mechanism of trans-placental dissemination of ZIKV remains unknown. Here, we sought to determine whether monocytes, which can cross tissue barriers, assist ZIKV dissemination to the fetus. We determined this by infecting monocytes with two strains of ZIKV: South American (PRVABC59) and Nigerian (IBH30656) and analyzing viral replication. We found that ZIKV infects and replicates in monocytes and macrophages, which results in the modulation of a large number of cellular genes. Analysis of these genes identified multiple pathways including inflammasome to be targeted by ZIKV, which was confirmed by analyzing the transcript levels of the proteins of inflammasome pathways, NLRP3, ASC, caspase 1, IL-1 and IL-18. Interestingly, IFNα and the IFN inducible gene, MxA were not enhanced, suggesting prevention of innate antiviral defense by ZIKV. Also, inhibition of inflammasome led to an increased transcriptional activity of IFNα, MxA and CXCL10. Based on these results we suggest that ZIKV transcription is regulated by inflammasomes.

## Introduction

In 2015, an unusually high number of newborns with microcephaly were reported in South America during Zika virus (ZIKV) outbreak^1^. Soon after, studies confirmed the association between ZIKV infection of mothers and abnormal fetal brain development^2^. Investigations using animal model verified the assumption that ZIKV infection of pregnant mice lead to the intrauterine growth restriction, microcephaly and fetal death^3–6^. Analysis of ZIKV distribution in tissues demonstrated the presence of virus exclusively in the brain of the fetus targeting neuronal precursors as well as immature neurons^7^. The replication of ZIKV causes neuronal death and brain deformity resulting in postpartum microcephaly.

ZIKV is typically self-limiting infection in most of the symptomatic adults. However, ZIKV has strong neurotropism, demonstrated in several animal studies^4–6^, which could explain the subsequent congenital neurologic sequelae in human. In the embryonic brain, ZIKV directly targets neuroprogenitors hampering cell differentiation and neurogenesis^4,6^. It appears that the apoptosis of neuroprogenitors is an ultimate cause of neurodegeneration. Increased expression of genes associated with autophagy and apoptosis is commonly found in the brains of pups born to dames infected with ZIKV^5,6,8,9^. In addition to virus replication related cell death, innate immune mechanisms were suggested to play role to control virus dissemination across placental barrier. Bayer *et al*. have shown that interferons (IFNs) produced by placental trophoblasts protect against ZIKV infection^10^. It appears that type I and type III IFNs are essential for protection against ZIKV, as animals with impaired IFN response are more susceptible to infection^11,12^. IFNs are produced by leukocytes including monocyte derived dendritic cells^13^. Therefore, it remains to be determined whether ZIKV infection upregulates type I IFN production in monocytes.

It is believed that ZIKV replicates in the epidermal keratinocytes at the site of the mosquito bite after infection^14^. Following 3–12 days post-infection, virus disseminates and can be detected in the blood, urine, semen and saliva^15–17^. According to the CDC, ZIKV viremia is present 24 hours after the onset of symptoms^18^, suggesting an early dissemination of the virus. In order to affect the neuronal development of the fetus, ZIKV has to cross the placental barrier to reach the fetus. One of the mechanisms utilized by viruses to cross the placenta is via leukocytes, where monocytes are often identified as the carriers^19–21^. Monocytes are a mobile population of leukocytes capable of crossing blood-tissue barrier^22^. They can cross tissue barriers, including the placenta and thus introduce pathogens to the sites that are normally protected from the infectious assault^22–24^. Human cytomegalovirus (HCMV), which causes congenital birth defects infects hematopoietic progenitor cells including monocytes to cross placenta and supports viral replication following differentiation into macrophages^25,26^. Since, ZIKV causes microcephaly (congenital birth defects), we speculate that ZIKV can also cross the placenta while carried by monocytes. However, little is known about monocyte susceptibility to ZIKV infection and also whether monocytes can support the replication of ZIKV.

Monocytes are sentinel leukocytes detecting pathogens and responding with an array of inflammatory cytokines, including IL-1 and IL-18^27^. An increased production of IL-1 and IL-18 by monocytes can enhance inflammation and significantly impact the outcome of the infection. Production of IL-1 and IL-18 is tightly regulated and involves an activation of inflammasomes^28^. The inflammasomes are an intracellular signaling complex containing NLRP3, pro-caspase 1 and the adaptor protein, ASC. Once assembled, inflammasomes release active caspase 1, which subsequently cleaves pro-IL1 and pro-IL18 producing active form of IL-1 and IL-18^29,30^. Both, IL-1 and IL-18 belong to the IL-1 superfamily of cytokines, which are characterized by their ability to control over inflammation and immune response, acting as potent activators of diverse cell types, such as macrophages, dendritic cells, neutrophils, B cells, T cells and endothelial cells^31–34^. Additionally, IL-1 family members have been implicated in polarization of an adaptive immune response toward Th1 (IL-18) and Th17 (IL-1) subset of T lymphocytes^32^. IL-18 was shown to activate the local T-regulatory lymphocytes, which is essential to maintain tolerance to self-antigens and inhibit proliferation of effector lymphocytes^35^. However, when overexpressed, IL-1 and IL-18 can cause severe life threatening inflammation^36,37^. Therefore, by production of IL-1 and IL-18, inflammasomes can regulate the severity of inflammation as well as the immune response.

Here, we show that two strains of ZIKV, South American (PRVABC59) and Nigerian (IBH30656) can infect monocytes and actively replicate, resulting in a transcriptional deregulation of cellular genes. Infection with both these strains of ZIKV led to a transcriptional deregulation of over 2,000 cellular genes in two independent experiments compared to the mock-infected monocytic, control cells. Interestingly, a large number of genes (1310 genes in set 1 and 1501 genes in set 2) were commonly deregulated with both the strains of ZIKV. Analysis of these commonly affected genes through iPathways analysis tool identified multiple pathways, including inflammasome as activated pathways in ZIKV infected monocytes. Activation of inflammasome pathway was confirmed by quantitative PCR, which detected an increased accumulation of NLRP3, ASC, caspase 1, IL-1 and IL-18 transcripts in ZIKV infected monocytes. Interestingly, infected monocytes did not show activation of IFNα and IFN inducible gene, MxA, suggesting that virus prevented activation of innate antiviral defense. Intriguingly, an inhibition of inflammasomes led to an increased transcriptional activity of IFNα and interferon inducible genes, MxA and CXCL10 suggesting that transcription of ZIKV depends on activation of inflammasome.

## Results

### Monocytes are susceptible to ZIKV infection

Majority of the CD14+ leukocytes in circulation are the monocytes^38^. Therefore, we wanted to determine whether ZIKV infects circulating CD14+ monocytes isolated from the human cord blood. The monocyte susceptibility to ZIKV infection and the efficacy of virus replication was analyzed by infecting the monocytes in two independent experiments (Set 1 and Set 2) and detecting the viral genome using next generation sequencing (NGS), qPCR, and viral proteins using Western blot and immunofluorescence assays.

**Figure 1:**
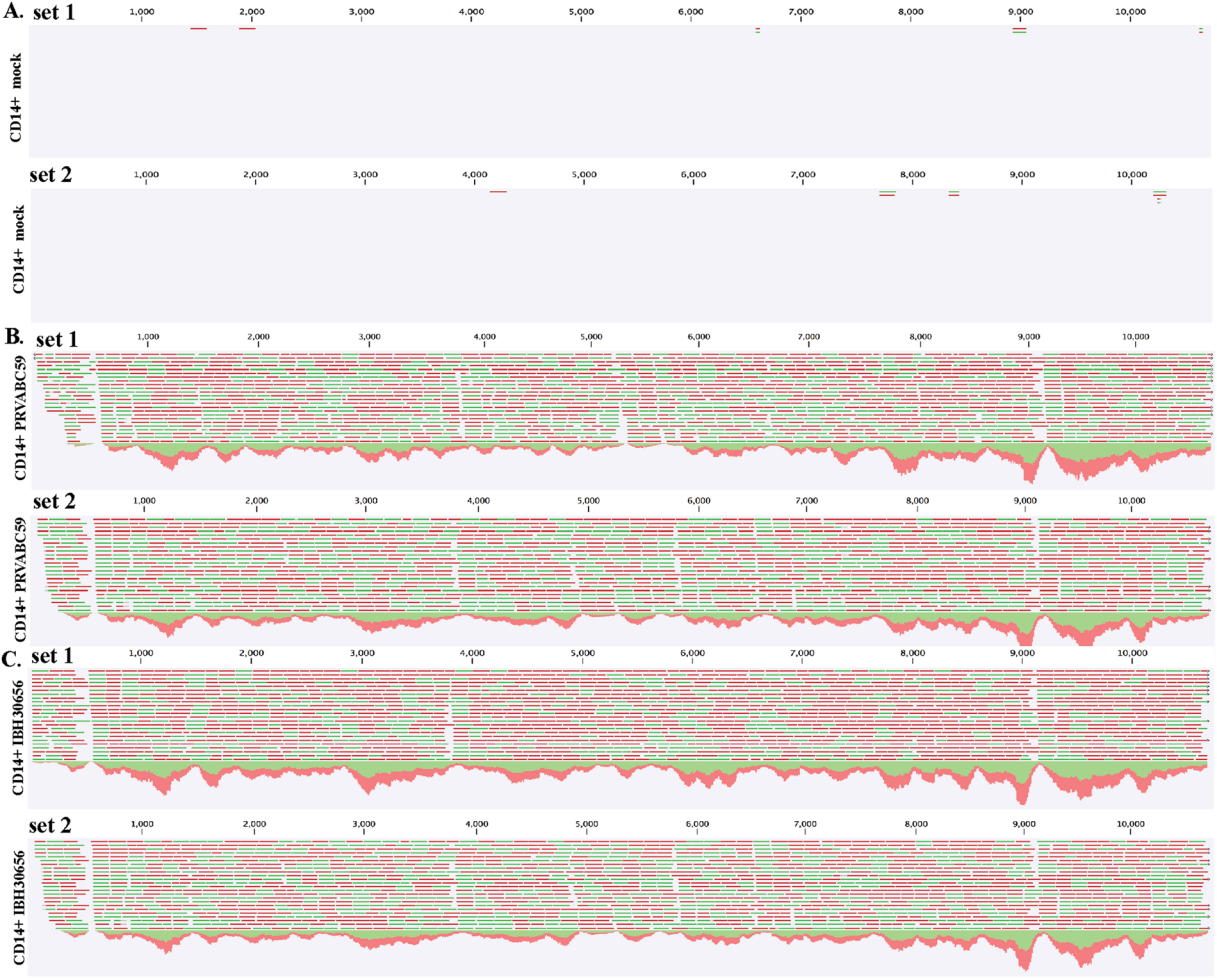
ZIKV transcripts were detected in infected monocytes by next generation RNA-seq analysis. (**A**) Mock infected CD14+ monocytes (12 hpi) were used for RNA extraction and RNA-seq analysis. RNA-seq data from two independent infection (set 1 and set 2) were used for mapping with ZIKV genome. (**B**) Mapping of RNA-seq reads from two independent infection of CD14+ monocytes (set 1 and set 2) with South American strain, PRVABC59 at 12 hpi. (**C**) Mapping of RNA-seq reads from two independent infection of CD14+ monocytes (set 1 and set 2) with Nigerian strain of ZIKV (IBH30656) at 12 hpi. Red and green lines represent the reads mapped to the viral genome, and the downwards peaks represent the sequence depth in each region.

**Figure 2:**
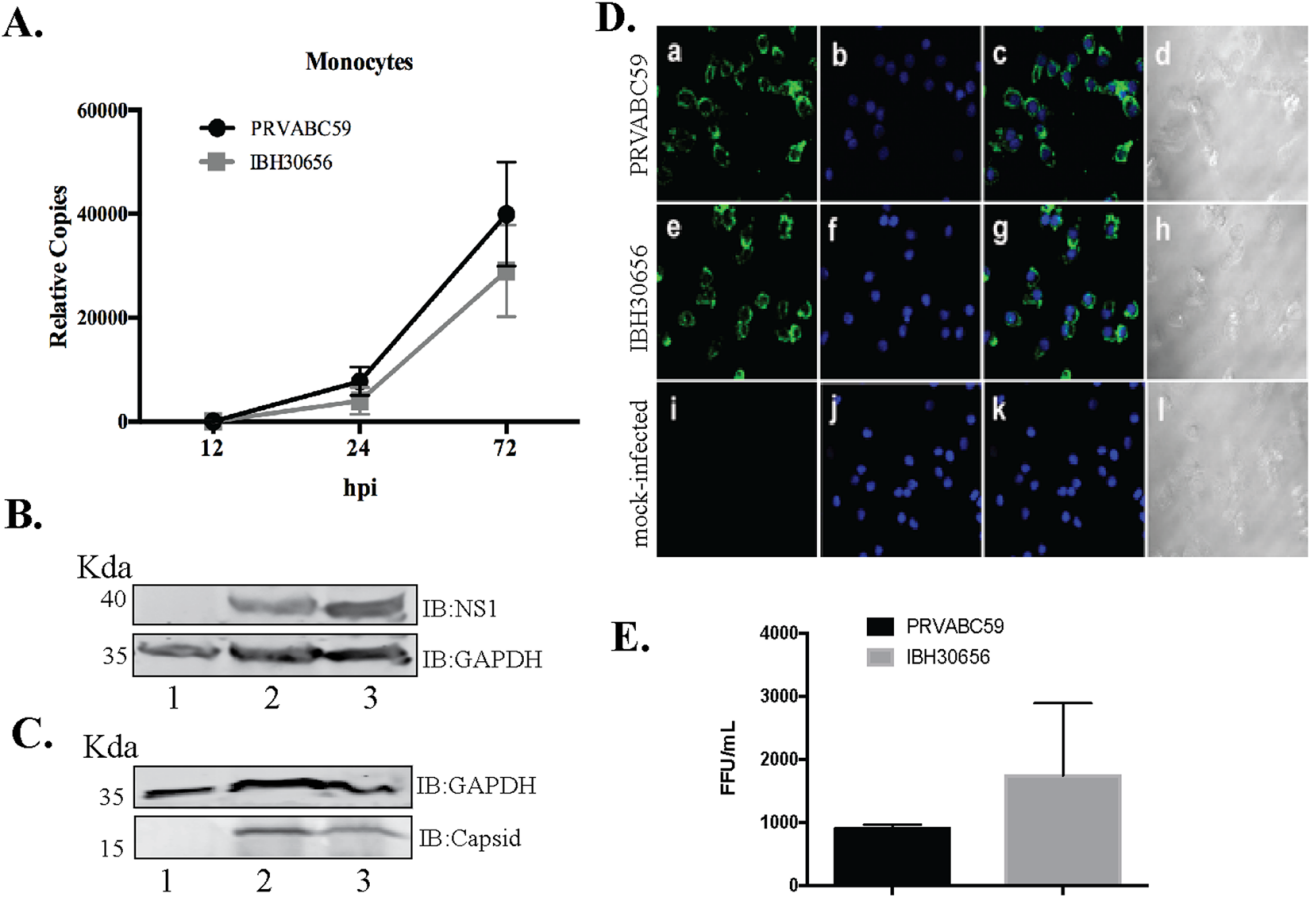
ZIKV actively replicates in the monocytes. Monocytes were infected with PRVABC59 (59) and IBH30656 (656) ZIKV strains. (**A**) Total RNA was collected at 12, 24 and 72 hpi for the detection of ZIKV, which was analyzed by qPCR by targeting a section of the viral genome coding for the envelop protein. Relative copies of the viral genomes (ZIKV) were calculated using ∆∆Ct method. (**B**, **C**) Western blot analysis of NS1 and capsid proteins in ZIKV infected monocytes. Monocytes were infected with PRVABC59 and IBH30656 ZIKV. At 24 hpi total proteins were collected and used for the detection of NS1 and capsid proteins. GAPDH was detected as the loading control. Lanes, 1-mock infected control monocytes, 2-monocytes infected with ZIKV strain PRVABC59 and 3-monocytes infected with ZIKV strain IBH30656. (**D**) Immunofluorescence analysis of ZIKV NS1 protein in the infected monocytes. Monocytes were infected with PRVABC59 and IBH30656 ZIKV. 24 hpi; monolayers were fixed and probed with rabbit anti-ZIKV NS1 antibody. Nuclei were stained with TO-PRO-3 (ThermoFisher, Inc.). Images were captured using Carl Zeiss LSM 780 confocal laser-scanning microscope. Monocytes infected with PRVABC59 ZIKV: a-NS1 localization, b-nuclei and c- merge of a and b, d-DIC. Monocytes infected with IBH30656 ZIKV: e-NS1 localization, f-nuclei and g- merge of e and f, h-DIC. Mock infected monocytes: i-NS1 localization, j-nuclei and k- merge of i and j, l-DIC. (**E**) Detection of virions in the supernatant of ZIKV infected monocytes. Plaque forming assay was used determining the infectious virus in supernatant. Supernatants from monocytes infected with PRVABC59 and IBH30656 ZIKV strains of ZIKV were collected at 72 hpi and used for inoculating Vero monolayer for 1 hour. Monolayers were overlaid with agarose (1%) containing DMEM culture medium. Seven days later, monolayers were fixed (1% paraformaldehyde) and stained with Neutral Red. The virus titer is presented as focus forming units (FFU).

RNA-seq data from monocytes infected with ZIKV revealed accumulation of viral transcripts and mapping those reads to the reference ZIKV genome showed that both ZIKV strains (PRVABC59 and IBH30656) can efficiently infect the monocytes (Fig. 1). Mapped RNA-seq reads (represented by red and green lines in panels B and C of Fig. 1) with reference genome showed uniform distribution of reads confirming comparable infectivity of monocytes with both ZIKV strains. Mock-infected control cells showed mapping of only few reads (possibly due to a limited similarity of the cellular genes with ZIKV) confirmed specificity of read mappings (Fig. 1A). Furthermore, we determined the accumulation of viral copies by extracting the total RNA at 12 h, 24 h and 72 h post-infection from the infected monocytes (Fig. 2A). Mock infected cells were used as a control for calculating the relative copies of the viral genome at different time post-infection. Replication of both strains, South American (PRVABC59) and Nigerian (IBH30656), of ZIKV showed almost similar pattern of steady increase in viral copies (Fig. 2A). We further asked whether an accumulation of viral copies led to the expression of viral proteins, which was confirmed by the detection of viral NS1 and capsid proteins in a Western blot and immunofluorescence assays. These data confirmed an expression and accumulation of NS1 and capsid proteins in monocytes infected with both the strains of ZIKV (Fig. 2B,C and D). Immune detection of NS1 protein in cells infected with PRVABC59 (Fig. 2D, panel a) and IBH30656 (Fig. 2D, panel e) demonstrated that both strains of ZIKV can infect the majority of monocytes. To confirm that ZIKV replication in infected monocytes produced virions, we determined presence of the virus in the culture supernatants by plaque forming assay. Supernatants from 72 hpi monocytes were used in plaque forming assays. South American strain, PRVABC59 produced approximately 900 plaques per ml of culture supernatant, whereas Nigerian strain, IBH30656 showed slightly higher number of plaques forming units (Fig. 2E). These data conclusively showed that ZIKV infection of monocytes leads to a replication of virus and expression of viral proteins.

### Monocyte-derived macrophages (MoMØ) are susceptible to ZIKV infection

**Figure 3:**
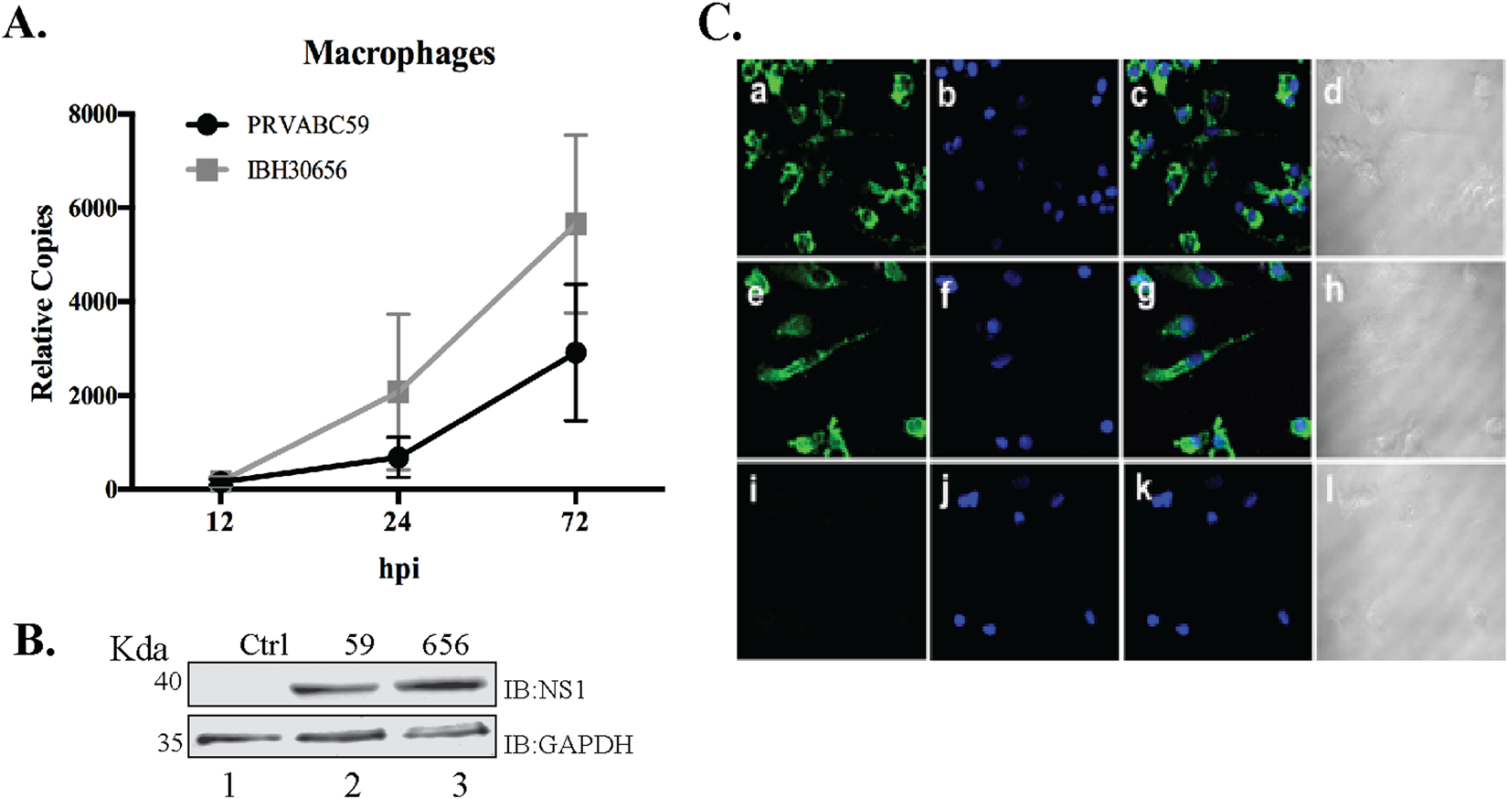
ZIKV actively replicates in the MoMØ. MoMØ were infected with PRVABC59 (59) and IBH30656 (656) ZIKV strains. (**A**) Total RNA was collected at 12, 24 and 72 hpi. Accumulation of ZIKV was analyzed by qPCR targeting section of the viral genome coding for the envelop protein. Relative copies of the viral genome (ZIKV) were calculated using ∆∆Ct method. (**B**) Western blot analysis of NS1 protein in ZIKV infected MoMØ. MoMØ were infected with PRVABC59 and IBH30656 ZIKV and total proteins were collected at 24 hpi for the detection of NS1 protein. Lanes, 1-mock infected control cells, 2- MoMØ infected with ZIKV strain PRVABC59 and 3- MoMØ infected with ZIKV strain IBH30656. (**C**) Immunofluorescence analysis of ZIKV NS1 protein in the infected MoMØ. MoMØ were infected with PRVABC59 and IBH30656 ZIKV. At 24 hpi; monolayers were fixed and probed with rabbit anti-ZIKV NS1 antibody. Nuclei were stained with TO-PRO-3 (Thermofisher, Inc.). Images were captured using Carl Zeiss LSM 780 confocal laser-scanning microscope. MoMØ infected with PRVABC59 ZIKV: a-NS1 localization, b-nuclei and c- merge of a and b, d-DIC. MoMØ infected with IBH30656 ZIKV: e-NS1 localization, f-nuclei and g- merge of e and f, h-DIC. Mock infected MoMØ: i-NS1 localization, j-nuclei and k- merge of i and j, l-DIC.

Upon infection, monocytes can differentiate into macrophages^39^. Also, the monocytes can differentiate into macrophages when they cross the blood-tissue barrier^40^. Therefore, we sought to determine whether monocyte will retain susceptibility to ZIKV infection after differentiation. MoMØ were derived by culturing monocytes in medium supplemented with GM-CSF (10 ng/ml). MoMØ susceptibility to ZIKV infection was analyzed by infecting them with both the ZIKV strains and analyzing the relative viral copies using qPCR and detection of viral protein, NS1 in a Western blot and immune localization assays. Relative quantitation of viral copies at 12, 24 and 72 hpi showed an increase in the viral copies (Fig. 3A). Interestingly, IBH30656 strain exhibited slightly higher viral copies compared to PRVABC59 in MoMØ (Fig. 3A). Replication of ZIKV in these MoMØ led to the expression of viral proteins and we detected NS1 through Western blotting and immunofluorescence. Anti-NS1 antibody detected specific protein in MoMØ infected with both the strains of ZIKV (Fig. 3B). Similarly, immunofluorescence assay with anti-NS-1 in these infected MoMØ showed abundant expression confirming replication of the virus (Fig. 3C). Collectively, these data support that MoMØ can be infected with ZIKV.

### **Analysis of the differentially regulated genes using iPathway tool**

**Figure 4:**
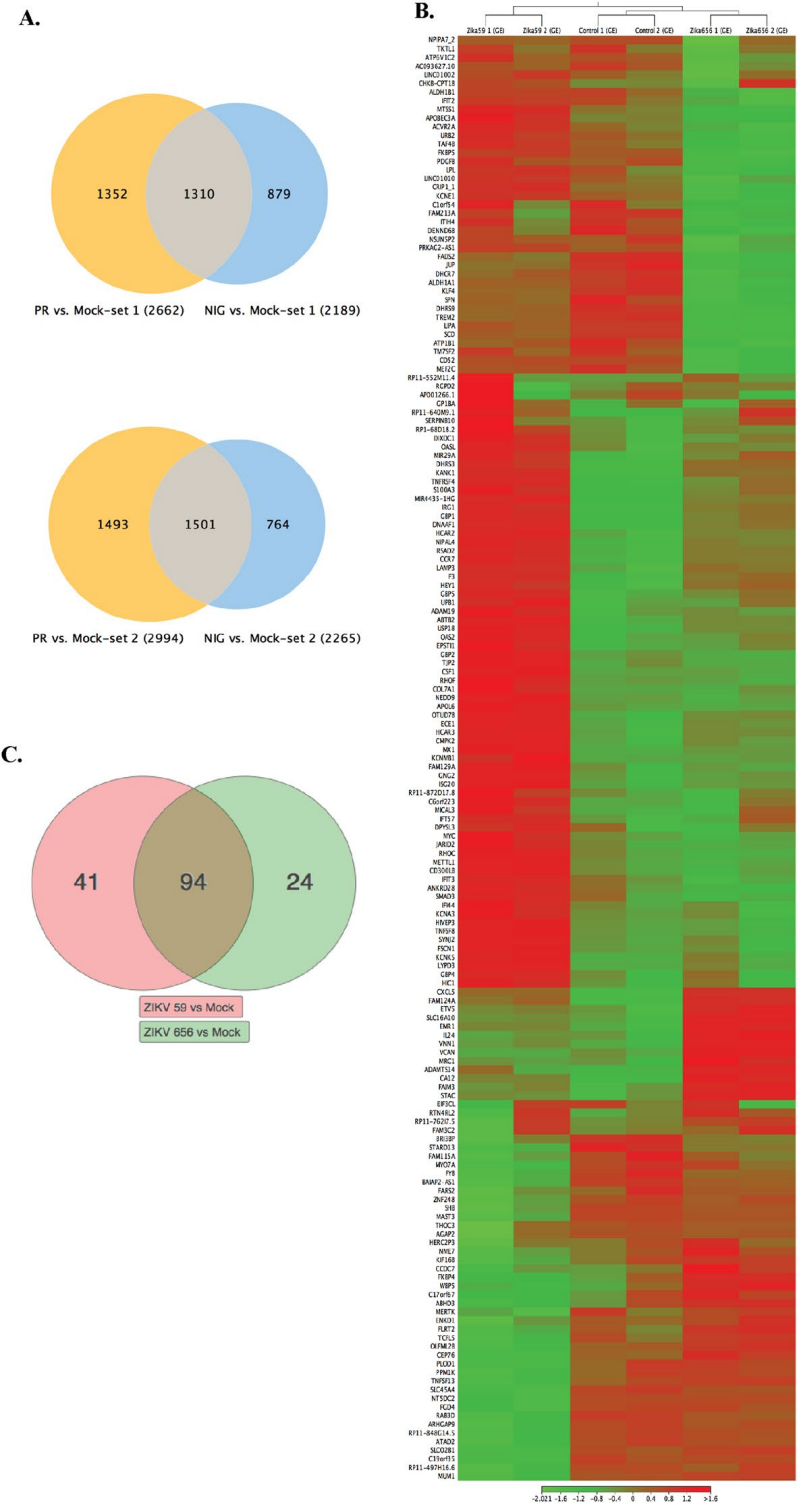
(**A**) Analysis of the cellular genes affected by ZIKV infection. (**A**) CD14+ monocytes infected with PRVABC59 (PR) or IBH30656 (NIG) were used for identifying deregulated genes due to ZIKV by comparing with the expression patterns of mock infected cells. Two independent infection and RNA-seq analysis (set 1 and set 2) were performed, which identified 2662 differentially expressed genes in PRVABC59 (PR) infected monocytes in set 1 and 2994 genes in set 2. Infection with IBH30656 (NIG) strain deregulated the expression of 2189 genes in set 1 and 2265 genes in set 2. Venn diagram generated by CLC workbench identified 1310 commonly deregulated genes in PRVABC59 (PR) and IBH30656 (NIG) infected monocytes in set 1 and 1501 genes in set 2. (**B**) Heat map showing the expression patterns of commonly affected genes by both the strains of ZIKV, PRVABC59 (59) and IBH30656 (656) in two independent sets (Set 1; Control 1, Zika 59 1, Zika 656 1 and set 2; Control 2, Zika 59 2, Zika 656 2). Red and green represents the upregulated and down regulated genes, respectively. High levels of similarity in the expression patterns of genes among set 1 and set 2 genes confirmed reproducibility of infection and next generation sequence analysis. (**C**) The deregulated genes were used for identifying the pathways affected by ZIKV using the iPathway analysis tool, which identified 94 common pathways by these two strains, PRVABC59 (ZIKV 59) and IBH30656 (ZIKV 656).

Cellular genes differentially expressed due to ZIKV infection were determined by mapping the RNA-seq data from 24 hpi to the human reference genome, hg19 using CLC Workbench 10.0.1. Relative expressions were calculated by determining the RPKM (Reads per kilobase of transcript per million mapped reads) of each cellular gene and comparing their RPKM values from the mock infected matched control CD14+ monocytes. Genes showing >2-fold deregulation (up and down regulation) in their RPKM values and <0.05 p-value, compared to the mock infected cells, were considered for our pathway analysis. Importantly, we saw a high level of reproducibility in the genes deregulated among two independent infection experiments. Two independent infection of CD14+ monocytes with PRVABC59 followed by RNA-seq analysis identified 2662 and 2994 differentially expressed genes in each assay (Fig. 4A). Similarly, the IBH30656 ZIKV infected CD14+ monocytes identified 2189 and 2265 differentially expressed in those two independent experiments (Fig. 4A). We found that a large proportion of differentially expressed genes were common among the genes affected by the ZIKV viral strains, PRVABC59 and IBH30656 (1310 genes in set 1 and 1501 genes in set 2). We also analyzed these differentially expressed common genes from PRVABC59 and IBH30656 infected cells using a heat-map analysis to determine the reproducibility in fold expressions among two independent experiments (Fig. 4B). Not surprisingly, majority of affected genes showed similar pattern in their expressions among the two independent experiments (Fig. 4B, compare Zika 59 1 with Zika 59 2, Control 1 with Control 2 and Zika 566 1 with Zika 656 2). This confirmed the reproducibility in our infection assays and differential gene expression. We further subjected these differentially expressed genes to an iPathways analysis for determining the cellular pathways, which were affected due to ZIKV infection. Our analysis identified 94 common pathways that were deregulated due to an infection of these two strains of ZIKV (Fig. 4C). Interestingly, PRVABC59 (ZIKV 59) had slightly higher number of uniquely regulated pathways (41) as compared to the IBH30656 (ZIKV 656), which had 24 uniquely affected pathways (Fig. 4C). Importantly, both the viral strains of ZIKV modulated the transcriptional levels of mitochondrial genes, although more pronounced effect was seen in the cells infected with South American stain, PRVABC59 as compared to the Nigerian strain, IBH30656. Interestingly, the mitochondrial genes (MT-ND2, MT-ND5 and MT-ATP8) activated in ZIKV infected monocytes are also shown to play an important role in the pathogenesis of other neuro-inflammatory neurodegenerative disease^28,41,42^. This may suggest that ZIKV targets the mitochondrial functions to induce pathogenesis.

**Figure 5:**
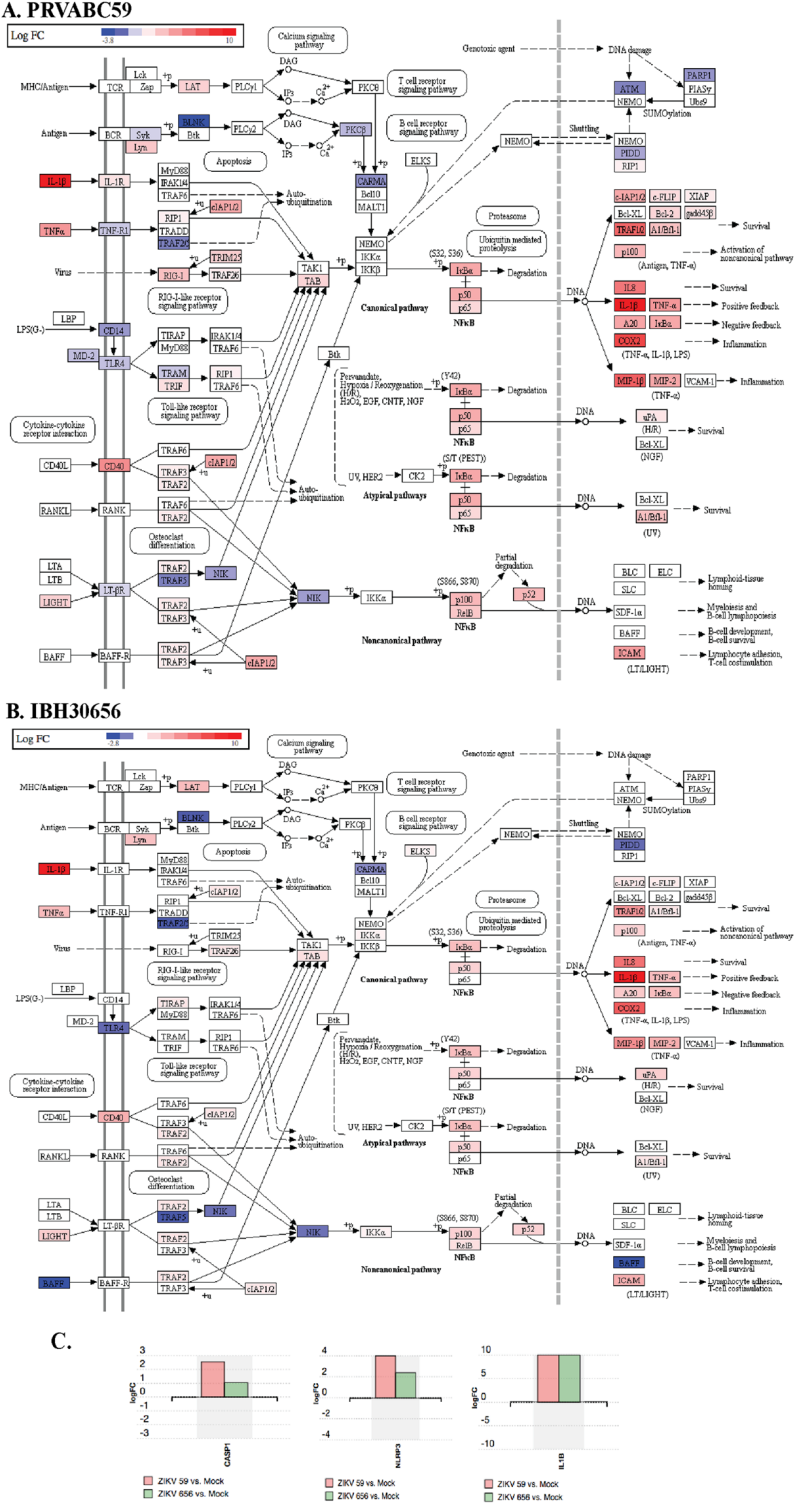
A representative pathway analysis generated by the iPathway tool shows genes affected by both strains of ZIKV in CD14+ monocytes. (**A**) Expression patterns of cellular genes in PRVABC59 infected monocytes. (**B**) Expression patterns of cellular genes in IBH30656 infected monocytes. Red and blue colored genes represent the up and down regulated genes, respectively (darker color represents a higher fold change compared to the mock infected cells). (**C**) Analysis of the inflammasome pathway genes, Caspase 1 (CASP1), NLRP3 and IL-1b) in RNA-seq data from the CD14+ monocytes infected with PRVABC59 (ZIKV 59) and IBH30656 (ZIKV 656).

Among the most deregulated pathways affected by ZIKV infection in CD14+ monocytes included apoptosis, chemotaxis, cell proliferation, inflammation, acute phase response and mitochondria activation. A representative pathway analysis is presented to show the names of the genes modulated by these two different strains of ZIKV (Fig. 5A, due to PRVABC59 and B, due to IBH30656). Interestingly, our analysis found that the majority of genes affected by both strains of ZIKV were common in each pathway (Fig. 5A,B, red represents an upregulation and blue a down regulation). Importantly, the intensities of blue and red, representing the fold change (darker color represents a higher fold change) were fairly similar among these two strains of ZIKV (Fig. 5B). Also, proteins involved in the inflammation pathways were highly affected by ZIKV (Fig. 5). Considering the importance of inflammasome pathway in viral replication and pathogenesis, we sought to pursue its role in ZIKV replication.

### Inflammasome activation in ZIKV infected monocytes

**Figure 6:**
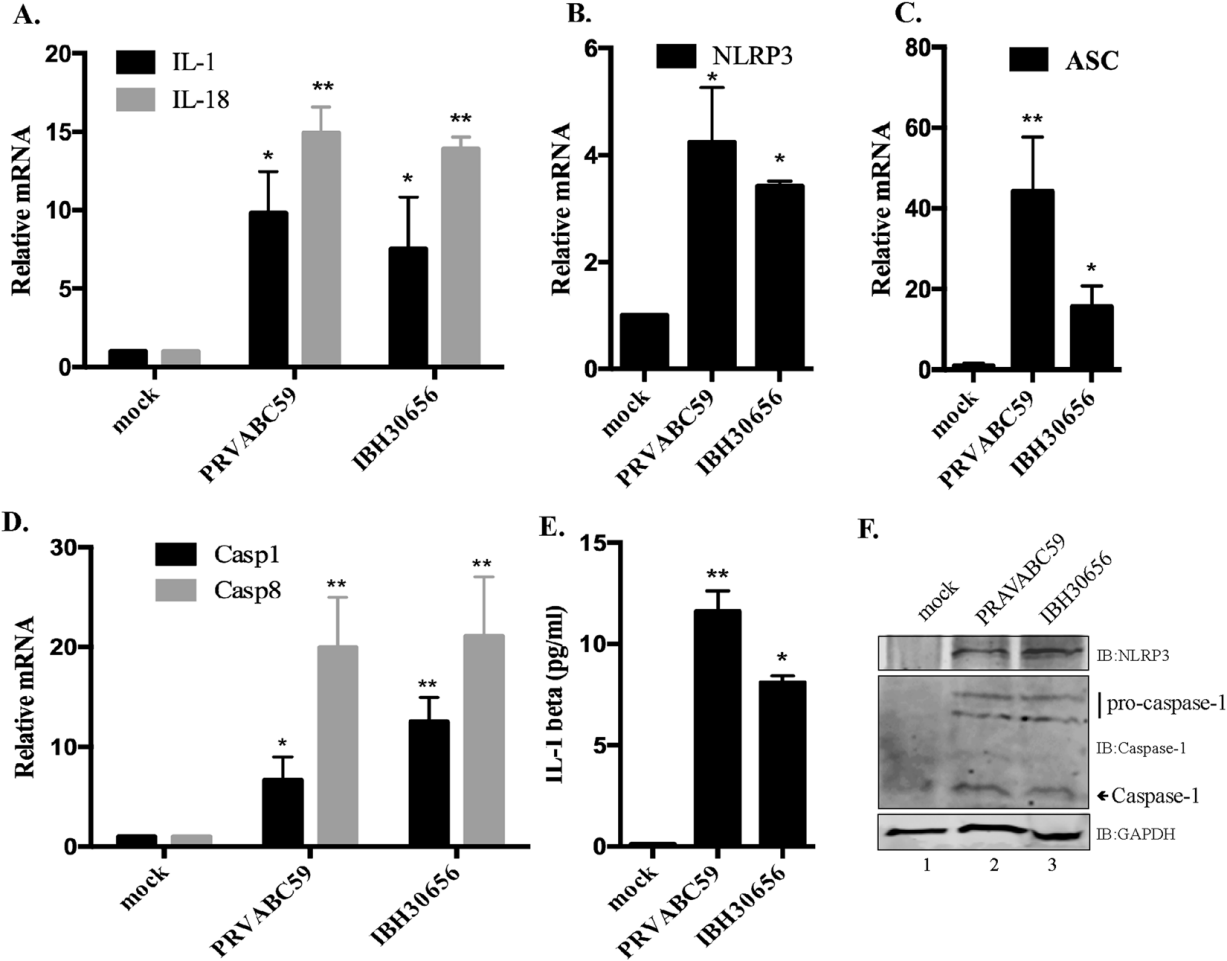
(**A**) qPCR analysis of IL-1 and IL-18 transcript levels in ZIKV infected monocytes. Total RNA was collected at 24 hours post infection.  Levels of IL-1 and IL-18 transcripts were analyzed by normalizing with GAPDH in corresponding samples. Relative levels for each gene were calculated in reference to mock infected cells using ∆∆Ct method. IL-1 dark bar, IL-18 grey bar. (**B**–**D**) qPCR analysis for the transcriptional levels of NLRP3, ASC, caspase1 and caspase 8. Total RNA was collected at 24 hours post infection. Transcriptional levels of NLRP3 ASC, caspase1 and caspase 8 were analyzed by qPCR. Ct values were normalized to the GAPDH of corresponding samples. Relative levels for each gene were calculated in reference to mock infected cells using ∆∆Ct method. (**E**) ELISA analysis of IL-1β levels in supernatant of ZIKV infected monocytes. Supernatants from mock-infected as well as ZIKV infected (strains PRVABC59 and IBH30656) monocytes were collected at 72 hpi. Levels of IL-1β were determined using Max Deluxe kit. The *P* values: *P < 0.05; **P < 0.01. (**F**) Western blot analysis of NLRP3 and caspase 1 expression in ZIKV infected monocytes. 24 hpi total proteins were collected and used for the detection of NLRP3, pro-caspase 1 and caspase 1. GAPDH was used as the loading control. Lanes, 1-mock infected control cells, 2-monocytes infected with ZIKV strain PRVABC59 and 3-monocytes infected with ZIKV strain IBH30656.

Inflammasomes are a complex structures which are assembled upon activation of pattern recognition receptors (PRR)^36^. Inflammasomes function to upregulate the innate immune response, facilitate inflammation and assist establishing T cell immune response^43^. There are several molecules including NLRP3 and pro-caspase 1, which become activated upon sensing viral RNA^44^. The final product of inflammasome activation is the release of an active form of IL-1 and IL-18 proteolitically cleaved from their inactive precursors^28^. To assess the activation of inflammasome pathway, transcriptional levels of caspase 1 (CASP1), NLRP3 and IL-1β were analyzed in the RNA-seq data collected from ZIKV infected monocytes. Expectedly, the levels of these inflammasome involved protein transcripts were upregulated when compared to the mock infected control cells (Fig. 5C). The levels of inflammasome involved proteins, NLRP3, ASC, caspase 1, caspase 8, IL-1 and IL-18 were further confirmed by quantitation of their transcripts in ZIKV infected monocytes. Our data showed that both the South American (PRVABC59) and Nigerian (IBH30656) strains of ZIKV increased transcript levels of IL-1 and IL-18 at 24 hours post infection, confirming an activation of inflammasome pathway (Fig. 6A). Supporting this conclusion, an increased transcriptional level of inflammasome key structural components, NLRP3, ASC and caspase 1, were detected in ZIKV infected monocytes (Fig. 6B–D). We also detected the production of IL-1β in ZIKV infected monocytes by an ELISA, which showed an increases levels of secreted IL-1β (Fig. 6E). Additionally, the protein levels of inflammasome pathway activators, NLRP3 and caspase1 were also detected, which was highly elevated in monocytes infected with both strains ZIKV. Interestingly, ZIKV infection induced the levels of both pro-caspase 1 and the cleaved caspase-1 (Fig. 6F).

Caspase 8 has also been shown to cleave pro-IL-1, thus promoting NLRP3 inflammasome response^45^. Therefore, we sought to determine whether ZIKV infection upregulates caspase 8 transcription in monocytes. Indeed, both strains of ZIKV led to a comparable upregulation in the levels of caspase 8 transcripts in monocytes (Fig. 6D). Since caspase 8 is also involved in the regulation of apoptosis, we suggest that elevated levels of caspase 8 due to ZIKV infection may contribute to the cell death via caspase 8 associated mechanisms.

### ZIKV infection inhibits IFNα transcription

**Figure 7:**
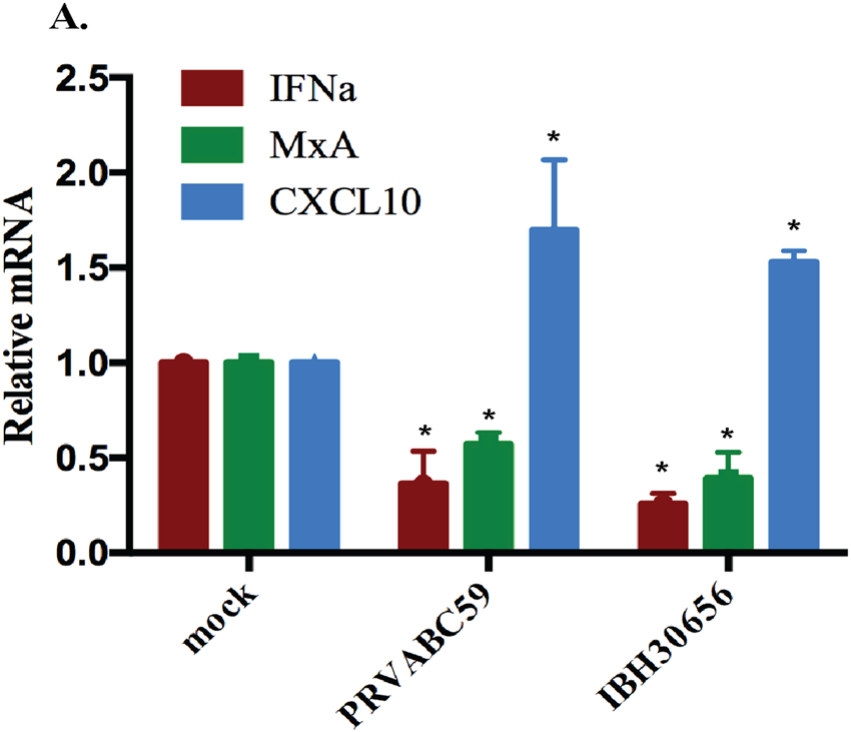
Effect of ZIKV infection on IFNa, MxA and CXCL10 transcription in monocytes. Monocytes were infected with PRVABC59 and IBH30656 strains of ZIKV. Total RNA was collected at 24 hours post infection. Accumulation of IFNα, MxA and CXCL10 transcripts was analyzed using qPCR. Real time PCR values were normalized to the GAPDH of corresponding samples. Relative levels of each gene were calculated in reference to mock infected cells using ∆∆Ct method. The *P* values: *P < 0.05; **P < 0.01.

Type I IFNs are typically produced by infected cells as a first step in anti-viral defense^46^. The early release of IFNs is essential for inhibition of viral replication via direct and indirect mechanisms. Therefore, we sought to determine whether ZIKV infection activates type I IFN transcription, which modulates the transcriptional activity of the IFN inducible genes such as MxA and CXCL10. We found that both ZIKV strains infection led to a reduced transcription levels of IFNα in monocytes (Fig. 7). Expectedly, the levels of IFN inducible gene, MxA was significantly reduced in ZIKV infected monocytes (Fig. 7). This may suggest that ZIKV averts activation of IFN dependent pathways to promote virus replication. Interestingly, the levels of CXCL10 was upregulated in ZIKV infected monocytes (Fig. 7), which could be due to the fact that CXCL10 does not pertain anti-viral activity but is better known as mononuclear leukocyte chemoattractant^47^. These observations led us to postulate that ZIKV infection could trigger mononuclear leukocyte migration towards the site of infection for ZIKV dissemination.

### ZIKV transcription depends on NLRP3 upregulation

**Figure 8:**
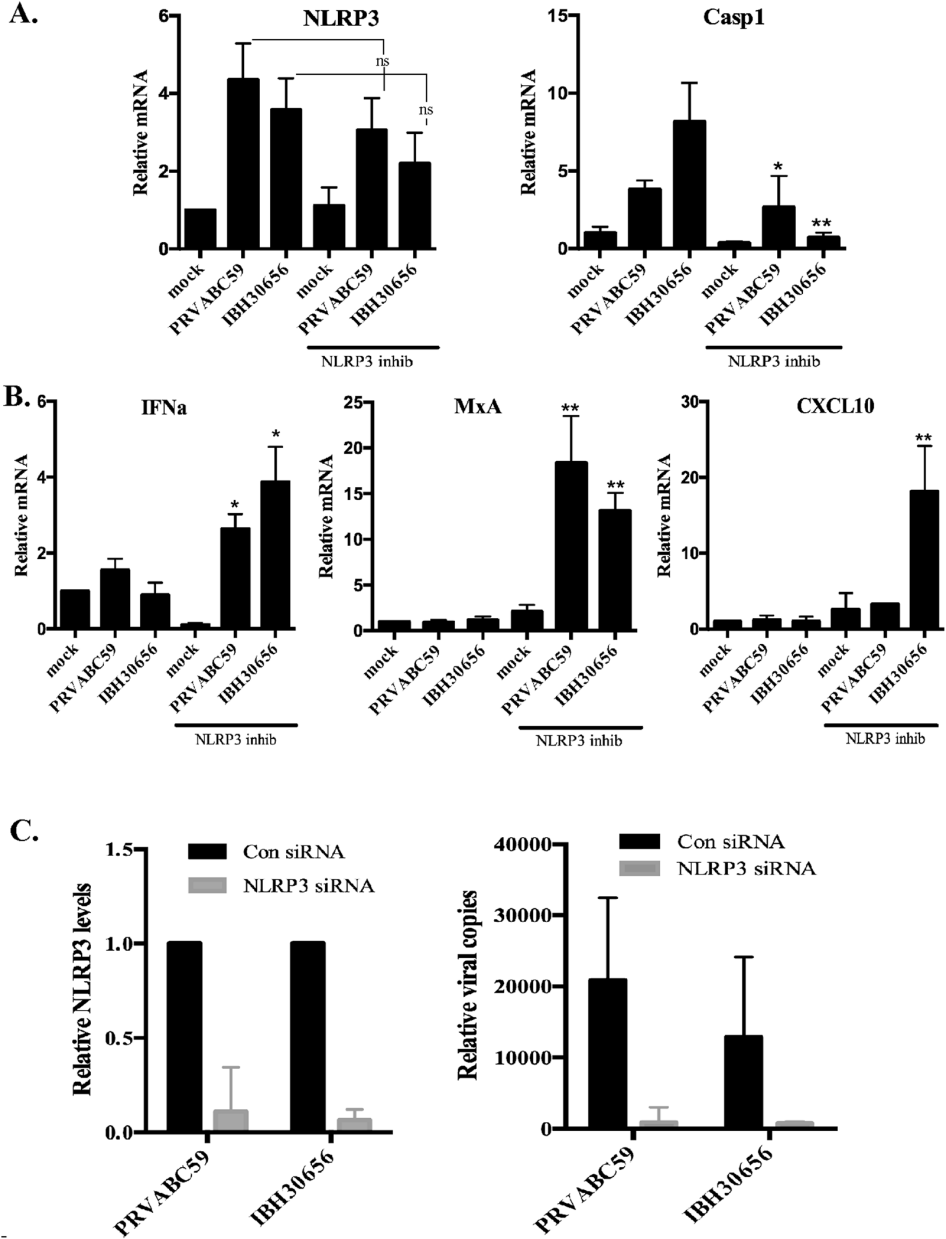
(**A**) Effect of isoliquiritigenin on NLRP3 and caspase 1 transcripts accumulation in ZIKV infected monocytes. Monocytes were incubated with isoliquiritigenin (10 μM; Sigma) for 3 hours prior to ZIKV infection. Total RNA was collected at 24 hours post infection. Accumulation of NLRP3 and caspase 1 transcripts were analyzed using qPCR. Real time PCR values were normalized to the GAPDH of corresponding samples. Relative levels for each gene were calculated in reference to mock infected and untreated cells using ∆∆Ct method. (**B**) Effects of isoliquiritigenin on MxA, CXCL10 and IFNa transcription in ZIKV infected monocytes. Monocytes were incubated with isoliquiritigenin (10 μM; Sigma) for 3 hours prior to ZIKV infection. Total RNA was collected at 24 hours post infection. Accumulation of MxA, CXCL10 and IFNa transcripts were analyzed using qPCR. Real time PCR values were normalized to the GAPDH of corresponding sample. Relative levels for each gene were calculated in reference to mock infected and untreated cells using ∆∆Ct method. The *P* values: *P < 0.05; **P < 0.01. (**C**) NLRP3 depletion reduced ZIKV replication in CD14+ monocytes. Monocytes were transfected with NLRP3 siRNA or control siRNA for 48 hours prior to infection. These cells were infected with both the strains of ZIKV and total RNA was collected 72 hpi. The level of NLRP3 was determined by detecting the mRNA levels, which showed a significantly reduced level in NLRP3 siRNA transfected monocytes as compared to the control siRNA transfected cells. ZIKV viral copies in NLRP3 si RNA or control siRNA monocytes were determined in a qPCR assay by normalizing with GAPDH of respective samples and comparing with mock infected cells using ∆∆Ct method.

Our data showed that ZIKV infection activates NLRP3 while inhibiting IFNα production in the monocytes (Figs. 6 and 7). To determine whether IFNα suppression is related to inflammasome activation, NLRP3 was inhibited by treating the monocytes with NLRP3 inhibitor, isoliquiritigenin (10 μM; Sigma) for 3 hours before infecting them with ZIKV. Total RNA was collected at 24 hours post infection for the detection of the transcript levels of NLRP3, caspase 1, IFNα and IFN inducible genes. Our data showed a slight reduction in the levels of NLRP3 transcripts in NLRP3 inhibitor treated cells (Fig. 8A). Additionally, the transcripts levels of caspase 1 were reduced in NLRP3 inhibitor treated cells, as expected (Fig. 8A). Importantly, inhibition of NLRP3 in ZIKV infected monocytes, led to a significant increase in the transcripts levels of IFNα and IFN inducible gene MxA (Fig. 8B). Interestingly, the levels of CXCL10 was upregulated in IBH30656 infected ZIKV but not with PRVABC59 ZIKV (Fig. 8B).

To confirm whether ZIKV activates NLRP3 to subvert antiviral response to promote viral replication, we depleted NLRP3 using siRNA approach and determined the levels of viral transcripts. Our data showed a significant reduction in levels of NLRP3 in siRNA NLRP3 transfected monocytes as compared to the control siRNA transfected cells (Fig. 8C). Total RNA from control siRNA and NLRP3 siRNA transfected CD14+ monocytes was used for the detection of ZIKV copies at 72 h post-infection, which showed a significantly reduced viral copies in NLRP3 siRNA transfected monocytes as compared to the control siRNA transfected cells. These assays were performed three independent times and the relative viral copies were calculated compared to the mock infected cells. This confirmed that specific inhibition of NLRP3 attenuates ZIKV replication.

## Discussion

ZIKV crosses the placenta reaching the fetus where neural tissue is mainly targeted^2^. Although the significant advances have been made in our understanding of the fetus brain pathology, the mode of virus trans-placental transmission remains unknown. Based on the fact that detection of ZIKV in the fetus coincides with the beginning of the maternal blood flow into the placenta^48^, we hypothesize that virus enters the placenta carried by leukocytes. Similar mechanism of trans-placental transmission has been shown for HIV-1 and cytomegalovirus^19,20^. Human Cytomegalovirus, another microcephaly causing virus crosses the placenta through infected monocytes, which differentiate into macrophages and supports viral replication in the fetus causing neuronal death^25,26^. Therefore, we believe that hematopoietic progenitor cells play an important role in fetal transmission of ZIKV and monocytes could serve as virus reservoir and disseminate virus to the fetus.

To understand the role of monocytes in ZIKV pathogenesis we sought to determine whether these cells are susceptible to the infection and can support virus replication. Our data revealed that South American (PRVABC59) and Nigerian (IBH30656) strains of ZIKV can infect and replicate in monocytes. Further replication of ZIKV led to an upregulation of a large number of cellular genes, which are involved in deregulation of multiple cellular pathways including apoptosis and cellular movement. The most striking observation was that upregulated genes were connected to pathogenesis of neuroinflammatory and neurodegenerative disease^42,49,50^. Additionally, some of the deregulated genes belonged to a group of genes involved in mitochondria functions, suggesting that ZIKV neuropathogenesis involves dysregulation of mitochondria. Therefore, we suggest that understanding the effects of ZIKV on the mitochondria function will provide more clues to the mechanisms involved in neurodegeneration.

Inflammasome pathway was also among the highly-deregulated pathways due to ZIKV infection in monocytes. Inflammasomes are complex structure, which cleave pro-caspase 1 releasing the functionally active caspase 1^51^. Caspase 1 proteolytically cleaves pro-IL-1 and IL-18, producing mature IL-1 and IL-18^30,52^. Both cytokines are known activators of inflammation and immune response^53,54^. To determine whether infection upregulated inflammasome, we investigated ZIKV caused changes in transcriptional activity of NLRP3 and caspase1, the main components of inflammasome complex. We, for the first time, have shown that infection with South American (PRVABC59) or Nigerian (IBH30656) strains of ZIKV activated NLRP3 and caspase 1 in monocytes. Additionally, increased transcriptional activity of IL-1 and IL-18 was found in monocytes infected with either strains of ZIKV. Therefore, we suggest that ZIKV infection causes inflammasome formation in monocytes. The significance of this observation is that inflammasomes are the source of powerful proinflammastory cytokines IL-1 and IL-18. Having IL-1 and IL-18 produced by monocytes is of great importance for ZIKV pathogenesis, because monocytes are mobile leukocytes capable of migrating from circulation into the tissue and crossing the blood tissue barriers. Therefore, monocytes with activated inflammasomes can produce inflammatory cytokines (IL-1 and IL-18) in the tissue, far from the original site of infection.

Monocytes play important role in regulating virus replication by producing IFNα^55^. IFNα establishes the “anti-viral” state by activating many IFN inducible genes including MxA and CXCL10^56,57^. MxA and CXCL10 control virus replication by targeting viral nucleocapsid^58^ or by activation of anti-viral immune response^59^. Our data show that ZIKV prevents IFNα activation in monocytes, supporting earlier observation made by Kumar *et al*.^60^. Furthermore, we found that ZIKV averts upregulation of MxA, the main antiviral protein targeting viral nucleocapsid protein^58^. Importantly, inhibition of inflammasome pathway by blocking the NLRP3 led to an activation of IFNα and IFN inducible proteins, MxA and CXCL10 in ZIKV infected monocytes. This is the first report showing involvement of inflammasome and IFNα pathways in ZIKV replication. Our data proposes a novel approach for the treatment of ZIKV infection by inhibiting inflammasome, NLRP3 protein.

### Conclusion

For the first time, we have shown that ZIKV infection activates inflammasomes in monocytes. Additionally, we have found that ZIKV infection represses the expression of IFNα and interferon inducible gene, MxA. These data suggest a mechanism of how ZIKV escapes IFN inhibition to establish long-term persistence. The most striking observation was that inhibition of inflammasome led to an accumulation of IFNα and IFN inducible genes in ZIKV infected cells. We believe that by inhibiting inflammsomes, IFN-dependent anti-viral state could be restored in ZIKV infected cells. Therefore, inflammasome could be a novel therapeutic target for the treatment of ZIKV infection.

## Materials and Methods

### Monocytes isolation

Cord blood buffy coats were obtained from the University of Colorado Cord Blood Bank, Aurora. Monocytes were separated using Miltenyi magnetic bead separation kit (CD14 Microbeads; Miltenyi, Auburn, CA). Cells were rested overnight and cultured in DMEM medium supplemented with 10% FBS (HyClone, Logan, UT), 2 mM l-glutamine, 25 U/ml penicillin, and 25 μg/ml streptomycin.

The purity of isolated human monocyte culture was examined by Flow cytometry using anti-CD14-FITC (Miltenyi, Auburn, CA) antibody.

Deidentified human cells were used in these assays and all the experiments were done in accordance with guidelines of the University of Nevada, Reno. The Environmental and Biological Safety committee of the University of Nevada, Reno, approved the methods and techniques used in this study.

### Macrophage differentiation

MoMØ were generated using culture medium supplemented with GM-CSF (10 ng/ml).

#### Virus, monocyte and MoMØ infection

Two ZIKV strains, PRVABC59 (South America) and IBH30656 (Nigeria) were obtained from ATCC (Manassas, VA). ZIKVs were propagated in Vero cells. Monolayers Vero cells were inoculated with each ZIKV strain for 2 hours. Unattached virus was removed by washing followed by adding fresh medium. Seven days later, virus was harvested, cell debris removed and virus was quantified by quantitative PCR and plaque forming assay.

Monocytes and MoMØ were incubated with each ZIKV (0.1 MOI) for 2 hours. At the end of infection, cells were washed three times and cultured in DMEM medium supplemented with 10% FBS (HyClone, Logan, UT), 2 mM L-glutamine, 25 U/ml penicillin, and 25 μg/ml streptomycin. Leukocytes were collected at indicated time points and stored at −80 °C until used.

#### Plaque forming assay for titrating ZIKV stocks

ZIKV titer was determined by plaque forming assay. Infected Vero cells were overlaid with agarose (1%) containing DMEM medium supplemented with 10% FBS, (HyClone, Logan, UT), 2 mM l-glutamine, 25 U/ml penicillin, and 25 μg/ml streptomycin. Seven days later, monolayers were fixed (1% paraformaldehyde) and stained with Neutral Red. Studies with ZIKV were conducted under the biosafety level 2+ (BSL2+) containment.

#### RNA extraction and Next Generation Sequencing (NGS)

Total RNA was extracted using Illustra RNAspin Mini kit (GE Healthcare, Marlborough, MA). The RNA was quantified using NanoDrop UV spectrophotometer (ThermoFisher, Waltham, MA) and quality of the RNA was determined using Bioanalyzer (Agilent Technologies, Santa Clara, CA). The poly-A containing mRNA was purified from 1 μg of total RNA. cDNA product was then used according to the TruSeq Stranded mRNA preparation guide (Illumina, San Diego, CA) to prepare the library. Library was validated, pooled, and normalized. Pooled libraries were diluted to a concentration of 4 nM, denatured with 0.2 N NaOH, and then diluted to 20 pM with Hybridization buffer (HT1). The denatured, pooled libraries were pipetted onto a MiSeq Reagent Kit v3 and loaded on a MiSeq according to the manufacturer’s recommended procedure. FastQ data generated by the MiSeq (Illumina, San Diego, CA) was annotated and the sequence reads were analyzed by CLC workbench 10.0.1 (Qiagen, Germantown, MD) for the detection of viral and cellular genes. Differential expressions of cellular genes expressions based on the RPKM (Reads Per Kilobase of transcript per Million mapped reads) were determined by comparing with mock-infected cells using RNA-seq analysis tool of CLC Workbench.

#### iPathway Analysis

Differentially regulated genes due to ZIKV infection, identified by CLC Workbench, were used to identify activated pathways using iPathway Guide (Advaita Corporation Inc.).

### Quantitative PCR (qPCR)

An aliquot of total RNA (40 ng) was used to synthesize the cDNA (Superscript kit; Invitrogen, Carlsbad, CA). cDNA (1 μl for each target) was used for the relative quantification of transcripts in a qPCR assay (ThermoFisher, Waltham, MA). Relative values were calculated by normalizing qPCR data to the respective GAPDH. The fold changes were calculated by ∆∆Ct method by taking the corresponding control group as reference. All qPCR reactions were performed at least in duplicates and repeated three times. The error bars represent standard deviation of three experiment replicates. Primer sequences are summarized in Table 1.

**Table 1:** Primers used for qPCR analysis.

Genes	Forward primer (5′-3′)	Reverse primer (5′-3′)
ZIKV	TTGGTCATGATACTGCTGATTGC	CCTTCCACAAAGTCCCTATTGC
Caspase 1	TGCCTTTCTTCTGGTCAGTG	TGCTGAGGTGAAGGAGAGAA
Caspase 8	TTTGCTTGTCTCTCGGTGTC	CTCGAACAGTACGCCACACT
NLRP3	ATGAGTGCTGCTTCGACATC	TTGTCACTCAGGTCCAGCTC
ASC	CGGATGAGCAGTACCAGGCA	GTCCAGTTCCAGGCTGGTGT
IL1	TGGACCTCTGCCCTCTGGAT	AAGGTCTGTGGGCAGGGAAC
IL18	AGGACAAGAGCCAGGAAGAA	ACTGCACCTTCACACAGAGC
MxA	CAATATGGAACCCAGCTCCT	CTAGAGGGACTCGTGTGCAA
IFNa	GGTTTAGGCTCACCCATTTC	GCCACCAGTAAAGCAAAGGT
CXCL10	GAGGAACCTCCAGTCTCAGC	AGAGGTACTCCTTGAATGCCA
GAPDH	CCATGTTCGTCATGGGTGTGAACCA	CACGATACCAAAGTTGTCATGGA

###  NLRP3 depletion using siRNA

Monocytes were transfected with either NLRP3 siRNA or control siRNA (Santa Cruz Biotechnology, Inc.) for 48 hours according to the manufacturer protocol. Briefly, 0.5 μg of siRNA was mixed with 5 μl of transfection reagent in final volume of 1 ml of transfection medium. Monocytes were incubated in transfection medium for 4 hours, washed and incubated in culture medium for 48 hours prior to infection.

### Enzyme-linked immunosorbent assay

IL-1β level in culture medium was determined using human IL-1β ELISA Max Deluxe kit (Biolegend; San Diego, CA) according to the manufacturer’s recommendations.

### Western blot

Total proteins were collected in 0.1% solution of sodium dodecyl sulfate (SDS) at 24 hours post infection. Proteins were separated on a 12% polyacrylamide mini gel (BioRad, Hercules, CA), transferred onto nitrocellulose membrane and blocked (5% non-fat milk in Tris-buffered saline (TBS) and 0.5% Tween 20) for 1 hour at room temperature. Membranes were then incubated (18 hours, 4 °C) with the rabbit anti-ZIKV NS-1 and Capsid protein polyclonal antibodies (1:5000; GeneTex, Irvine, CA), anti-GAPDH (1:2000; US Biological, Tustin, CA), anti-caspase 1 (1:200; Santa Cruz Biotechnology, Inc.) or anti-NLRP3 antibody (1:500; Santa Cruz Biotechnology, Inc.). The blots were washed (3x, TBST) and incubated with appropriate secondary antibodies conjugated with Alexa Fluor 680 (1:10,000; Molecular Probes, Carlsbad, CA). The membranes were scanned with the Odyssey scanner (Li-Cor, Lincoln, NE).

### Immunoflourescence analysis

ZIKV infected monocytes and MoMØ as well as mock-infected cells were fixed (3:1 methanol:acetone) and stored at −80° until used. Slides were permeabilized with 0.1% Triton X-100 for 30 min, washed (3x) and blocked (3% normal donkey serum, 0.5% BSA) for 60  min at room temperature. Washed again (3x) monolayers were incubated with rabbit anti-ZIKV NS1 polyclonal antibody (1:1000; GeneTex, Irvine, CA) primary antibody for 60 min at room temperature followed by incubation with goat anti-rabbit Alexa Fluor 488 (1:5000; Molecular Probe, Carlsbad, CA) secondary antibody for 60 min at room temperature in the dark. Finally, nuclei were stained with TO-PRO-3 (ThermoFisher, Walthman, MA). Slides were examined using Carl Zeiss LSM 780 confocal laser-scanning microscope.

#### Statistical analysis

Statistical analyses were performed using Prism 6.0 software (Graphpad Inc.) and the p-values were calculated using two-tailed t-tests. Asterisks represent the *P* value < 0.05 (*) and *P* value < 0.01 (**).

### Genebank accession number

The RNA-seq data is submitted to Gene Expression Omnibus (Accession number GSE103114) and can be accessed by the following link- https://www.ncbi.nlm.nih.gov/geo/query/acc.cgi?acc=GSE103114.
